# Synthesis and Electrochemical Property of FeOOH/Graphene Oxide Composites

**DOI:** 10.3389/fchem.2020.00328

**Published:** 2020-04-30

**Authors:** Xingying Chen, Yanyang Zeng, Zehua Chen, Shuo Wang, Chengzhou Xin, Lixia Wang, Changliang Shi, Liang Lu, Chuanxiang Zhang

**Affiliations:** ^1^School of Medicine, Henan Polytechnic University, Jiaozuo, China; ^2^College of Computer Science and Technology, Henan Polytechnic University, Jiaozuo, China; ^3^College of Chemistry and Chemical Engineering, Henan Polytechnic University, Jiaozuo, China; ^4^School of Materials Science and Engineering, Tsinghua University, Beijing, China

**Keywords:** lithium-ion batteries, FeOOH, FeOOH/GO, anode materials, electrochemistry performance

## Abstract

A facile ultrasonication method was used to uniformly mix nanospindle-shaped FeOOH (80–100 nm) and a conductive matrix of graphene oxide (GO) to form FeOOH/GO composites. No carbon peak was observed in the X-ray diffraction pattern, indicating that the graphene oxide did not stack together and that the dispersion of graphene was very high. X-ray photoelectron spectroscopy (XPS) tests showed that the formation of Fe-O-C bonds played a positive role in electron transport, revealing that it has a certain impact on the electrochemical performance of FeOOH/GO. The FeOOH/GO was further characterized by TGA, and the content of GO in the synthesized sample was 6.68%. Compared with that of FeOOH, the initial discharge capacity of FeOOH/GO could reach 1437.28 mAh/g. Additionally, compared to that of pure FeOOH, the reversibility of the electrochemical reaction of FeOOH/GO was improved, and the impedance value was reduced. Finally, FeOOH/GO was used directly as a lithium-ion battery (LIB) anode material to improve the kinetics of the Lithium ions insertion/extraction process and improve ionic conductivity.

## Introduction

Nowadays, electrochemical secondary batteries are a promising technology because of their high energy conversion efficiency (Marcano et al., 2010; Etacheri et al., 2011; Vikström et al., 2013; Han et al., 2014; Deng, 2015; Song et al., 2016). Lithium-ion battery (LIB) is considered an important energy storage system. Because of its long cycling life and high energy density, it can be widely used in electric vehicles, large-scale power grids and portable devices. In a battery system, graphite, as a commercial anode material, has a theoretical capacity of only 372 mAh/g (Lin et al., 2019). Coupled with its limited rate performance, graphite is expected to have difficulty meeting the future requirements of high-power batteries. Therefore, the development of new anode materials is an effective way to solve the above problems.

The iron oxide family (Fe_2_O_3_, Fe_3_O_4_) is considered a candidate material for the next generation of lithium-ion anode materials (Chen et al., 2019a,b; Li et al., 2019). Iron oxide has the following advantages: easy availability, natural nontoxicity, low cost and very high theoretical capacity. FeOOH is a kind of material similar to iron oxide, and thus, FeOOH has similar performance and electrochemical activity as iron oxide. Amine first reported that FeOOH has superior cycling performance (Amine et al., 1999). Xu found that FeOOH has a capacity of 905 mAh/g (Yu et al., 2015a). This has led to further explorations of the cycling and rate performance of FeOOH. However, previous works have not found a good solution for the conductivity of FeOOH. In addition, FeOOH has similar physical and chemical properties as iron oxide, whose conductivity is also poor (Jung et al., 2013). Therefore, improving the conductivity of FeOOH is key to improving the capacity of FeOOH. At present, the reported method to improve the conductivity of FeOOH is mainly to increase the amount of a conductive agent when preparing electrode sheets; the conductive agent amount can reach up to 40%. Therefore, many conductive agents are bound to increase the volume of electrode materials and reduce the capacity provided by unit volume. It has been reported that the conductivity and lithium-ion diffusion rate of many materials can be improved by compounding with graphene. In recent years, the formation of composites has been an effective method to improve the conductivity and stability of electrode materials. It is often used in alloy materials, and in metal oxide and sulfide battery systems to improve the performance of lithium and sodium storage (Liu et al., 2016; Zhang and Du, 2016; Wei et al., 2017). Usually, carbon materials such as graphene, carbon nanotubes and three-dimensional foam carbon can be used to provide efficient conduction paths to improve the electrical conductivity and structural stability of electrode materials (Cao et al., 2014; Xu et al., 2016; Guo et al., 2017; Lu et al., 2017). However, there are few reports on how graphene can promote the property of active materials. In a charge/discharge cycle, the mechanism that graphene promotes during the electrochemical reaction under high current, and the difference between a graphene chemical composite and a physical mixture should be investigated (Yu et al., 2015a; Zhai et al., 2015). Therefore, more details of graphene composites are worth exploring. Only when the above questions are explained clearly can the performance of active materials be more effectively improved by compounding with graphene (Sun et al., 2013).

In this work, FeOOH is grown on a graphene substrate. The composite structure of FeOOH/GO is controlled by adjusting the synthesis process to improve the binding force between them as well as the lithium storage performance of the composite.

## Experimental Section and Characterization

Hydrothermal method was used. Briefly, 9.050 g of FeCl_3_ and 1.216 g of lysine were added to 166.7 ml of deionized water and stirred for 2 h. The hydrothermal reaction is at 130°C in stainless steel reactor for 10 h. Next, the hydrothermal reaction product was washed until it reached a pH of 7. After drying overnight in a vacuum oven at 110°C, the FeOOH particles were finally obtained. FeOOH/GO was synthesized using the same method with the following raw materials: 9.050 g of FeCl_3_, 0.2 g of GO and 1.216 g of lysine. The raw materials were subjected to ultrasonication for 8 h before the hydrothermal reaction. Finally, the synthesis method of GO was based on Hummers' reported (Marcano et al., 2010; Gogotsi, 2012).

The crystal structure and morphology were characterized by Hatchi SU8020, and XRD test was performed on SmartLab using Cu Kα (λ = 1.54178 Å) radiation at 40 kV. X-ray photoelectron spectroscopy (XPS) was further implemented with the FeOOH/GO composite to research the chemical states of all the bonded elements at the surface.

LIR2016 coin-type half-cells with FeOOH as the active material of the negative electrode, a Celgard 2250 film as a separator, lithium metal as a counter electrode and N-methylpyrrolidone as a solvent were fabricated to evaluate the electrochemical performance using LANHE (CT2001A). The synthesized active material, conductive carbon black and polyvinylidene fluoride were mixed with a mass ratio of 80:10:10. Then the mixture dissolved in a solvent to form a slurry. The slurry was scratched on Cu foil. And then dried at 120°C for over 10 h in air. The commercial electrolyte was purchased from Guotai-Huarong Co. Ltd., Jiangsu, China. The volume ratio of EC to DMC was 1:1. A lithium sheet was used as the counter electrode and reference electrode when assembling the half cell. The voltage range of the constant current charge and discharge test was 0.01 ~3.0 V. The specific capacity of the charge and discharge was calculated per unit mass of the active powder. Cyclic voltammetry (CV) measurements were tested by CHI 650D (ChenHua, Shanghai) between 3.0 and 0.01 V vs. (*Li/Li*^+^) at scan rates of 0.1, 0.2, 0.5, 1, and 2 mV/s. Electrochemical impedance spectroscopy was performed on electrochemical workstation (Parstat 2273, Princeton) in a range of 0.1 Hz to 50 kHz with an amplitude of 10 mV.

## Results and Discussion

**Figure 1:**
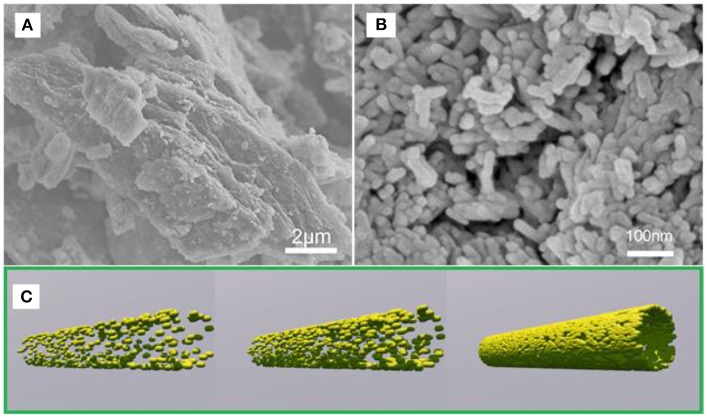
SEM image of FeOOH **(A)** and FeOOH/GO **(B)**. **(C)** Schematic showing the synthesis of FeOOH/GO.

Figures 1A,B shows the SEM images of FeOOH/GO, illustrating that FeOOH is a spindle-shaped structure with a length of 80-100 nm and a diameter of <10 nm. Additionally, FeOOH was attached to nanoscale GO, and the FeOOH and GO were well-connected. The morphology of the pure FeOOH and FeOOH/GO composites was characterized by SEM. As shown in Figures 1A,B, the prepared pure phase of FeOOH has a block structure. However, the FeOOH on the graphene composite shows a rod-like structure with a particle size <200 nm, indicating that FeOOH nucleation on the surface of graphene affects the growth of FeOOH. After the hydrothermal reduction and ultrasonication, the size of graphene and FeOOH decreased significantly (Wang et al., 2013). The epoxy groups on the surface of graphene oxide aggregated in a linear arrangement. These linear arrangements of epoxy groups were finally removed in the form of CO_2_, which formed a linear arrangement of defect aggregation or cracks on the surface of the graphene oxide. After ultrasonic treatment, the graphene oxide broke along the linear defects, resulting in small nanoparticles (Yang et al., 2008). Figure 1C provides a schematic of the synthesis of FeOOH/GO.

**Figure 2:**
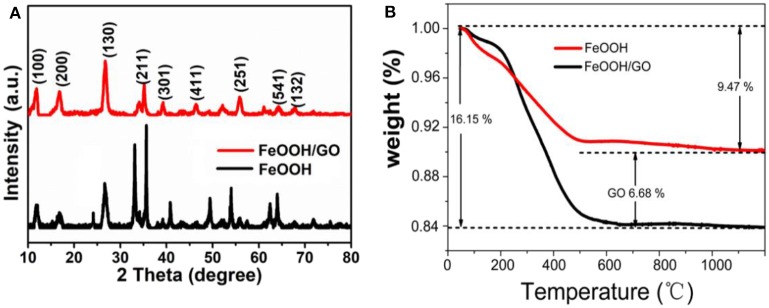
**(A)** X-ray diffraction patterns of the both samples. **(B)** Thermogravimetric Analysis curves of the both samples.

Figure 2A shows the XRD patterns of the both samples. All of the diffraction peaks were found to agree well with iron oxide hydroxide (scalable) (JCPDS No. 75-1594). Almost no peaks of any other phases were obtained. No carbon peak was observed in XRD, which indicated that graphene oxide did not stack together, and thus demonstrated a high dispersion after the attachment of FeOOH. The peak intensity of the FeOOH/GO XRD spectrum was lower than that of FeOOH, and broadening occurred, indicating that the particle size of the modified material was smaller (Zhou et al., 2014). Figure 2B is the thermogravimetric diagram of the sample. The diagram showed that the weight loss of pure FeOOH was 9.47% in a range of 100–500°C, which is close to the theoretical value for the thermal decomposition of FeOOH (10%). Figure 2B shows two obvious weight loss stages for the FeOOH/GO sample, which are mainly attributed to the dehydration of FeOOH and GO oxidation to CO_2_. By calculating the two weight-loss peaks, the contents of GO and FeOOH in the composite were found to be 6.68 and 93.32%, respectively.

**Figure 3:**
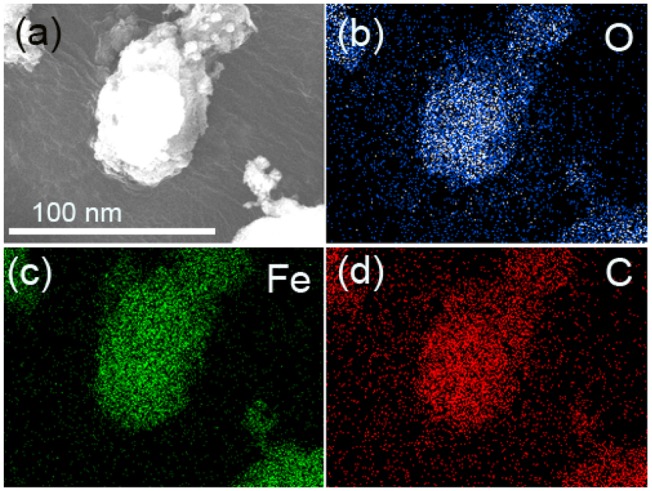
**(a)** TEM image of FeOOH/GO. **(b–d)** Elemental mappings of O, Fe, and C via energy-dispersive X-ray (EDX) spectroscopy.

Figure 3 presents the TEM diagram of FeOOH/GO. Figures 3b–d show the elemental mappings of O, Fe, and C, respectively, which were obtained by energy-dispersive X-ray (EDX) spectroscopy. In Figure 3, it showed that there were only three elements in the FeOOH/GO: Fe, O, and C.

**Figure 4:**
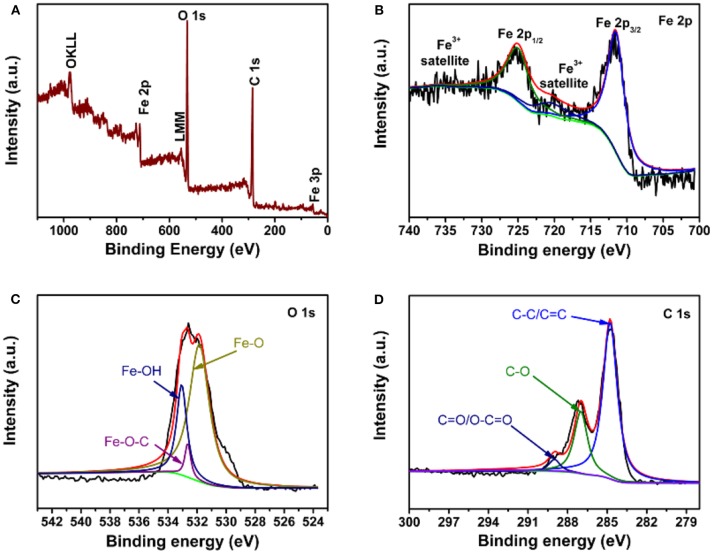
XPS of FeOOH/GO: **(A)** XPS survey scan spectrum; **(B)** Fe 2p core-level spectra; **(C)** O 1s core-level spectra; and **(D)** C 1s core-level spectra.

The relationship between the FeOOH rod and graphene was further studied by XPS. Figure 4A shows the full spectrum, indicating the existence of Fe, O and C. In Figure 4B, there were three peaks located at 711.4, 724.9, and 720 eV in the elemental spectrum of Fe 2p, which were satellite peaks of Fe 2p3/2, Fe 2p1/2, and Fe 2p3/2, respectively. There were three peaks located at 711.4, 724.9, and 720 eV in the elemental spectrum of Fe 2p, which were satellite peaks of Fe 2p_3/2_, Fe 2p_1/2_, and Fe 2p_3/2_, respectively; the above peaks agreed well with the results of a previous report for FeOOH (Chen et al., 2014). Figure 4C further confirms the existence of Fe-O-C bonds by combining the elemental spectra of O. The O 1s peaks could be divided into three peaks: Fe-O (531.2 eV), Fe-O-C (532.4 eV), and Fe-OH (533.1 eV) (Zou et al., 2014; Yu et al., 2015b). The Fe-O-C peak at 532.4 eV indicated the existence of an Fe-O-C bond between FeOOH and GO. Figure 4D shows the carbon spectra. The C 1s peaks showed that there were three valence bonds: C-C (284.1 eV), C-O (286.6 eV) and C = O (288.7 eV) (Yu et al., 2015c; Qi et al., 2016). The C-O bond may be C-O-H, and C-O-C and Fe-O-C bonds were found to exist in the FeOOH/GO complex (Sun et al., 2013). The core-level spectra of C 1s were similar to that of pure GO.

**Figure 5:**
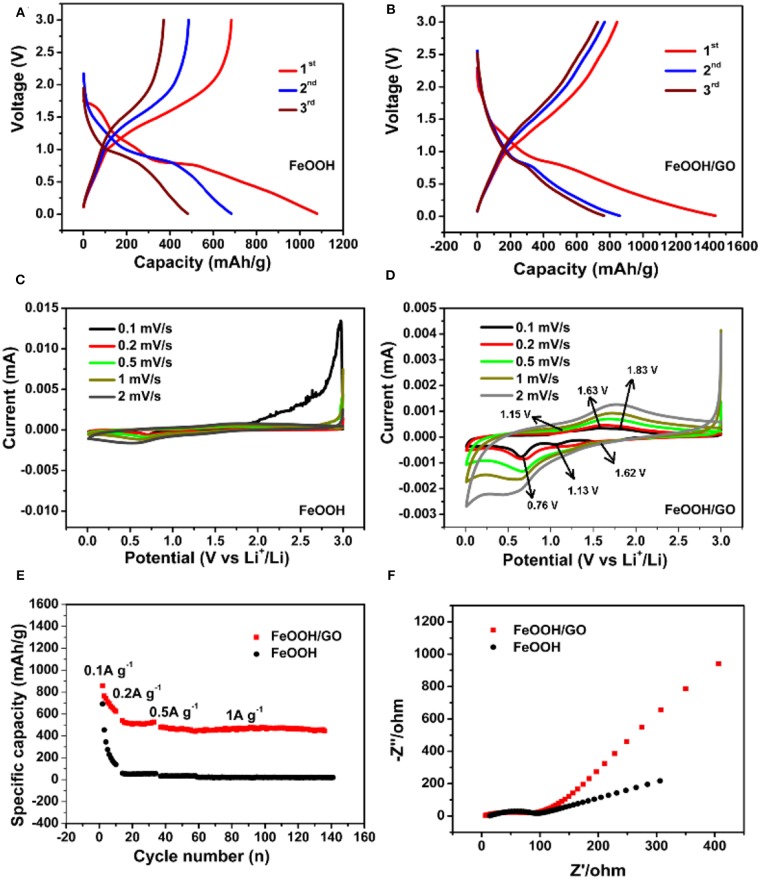
Galvanostatic discharge/charge curves of FeOOH **(A)** and FeOOH/GO **(B)** at a current density of 0.1 A/g. CV curves of FeOOH **(C)** and FeOOH/GO **(D)**. Specific capabilities of FeOOH and FeOOH/GO at different current densities **(E)**. EIS of FeOOH and FeOOH/GO **(F)**.

The charge discharge curves of the FeOOH/GO composite and FeOOH are shown in Figures 5A,B, respectively. There were two plateaus at ~ 1.27 and ~ 0.90 V in Figure 5B, representing the embedding reaction of Li_x_FeOOH and the conversion reaction of Fe(0), consistent with the information reflected in the CV curve in Figure 5D. The initial capacity of the FeOOH/GO composite reached 1437.28 mAh/g, which was higher than that of FeOOH (1079.21 mAh/g). The intercalation and deintercalation behaviors of the pure FeOOH and FeOOH/GO composites were studied by CV. In Figure 5C, the CV of the FeOOH electrode shows that the redox peak in the electrochemical process of FeOOH was not very obvious compared with that of the FeOOH/GO electrode. In Figure 5D, there were three reduction peaks at 1.62, 1.13, and 0.76 V, indicating that lithium intercalation was a multistep reaction. In Figure 5D, the peak at 1.62 V may be attributed to the transformation from FeOOH to Li_x_FeOOH (x < 1). The peak at 1.13 V represented the peak of Li_1+x_FeOOH (Zhai et al., 2016). The reduction peak at 0.76 V was related to the formation of metallic Fe and solid electrolyte interphase (SEI) films. In the next scan, the oxidation peak at 1.15 V belonged to the peak generated by the oxidation reaction during the decomposition of the SEI films. The oxidation peak at 1.63 V represented the transformation peak from Fe(0) to Li_1+x_FeOOH, and the oxidation peak at 1.83 V represented the reaction peak of Li_1+x_FeOOH to Li_x_FeOOH (Imtiaz et al., 2018). In the second CV cycle, most of the peaks were consistent with those in the first cycle, which showed that the conversion reaction was highly reversible. In contrast, the reaction peak of pure FeOOH was weak, reflecting poor reversibility. Figure 5E shows the rate performance of the both samples (current density from 0.1 to 1.0 A/g). In Figure 5E, the FeOOH/GO composites showed stable and high capacities at different current densities. At room temperature, the capacity reached 1437.28, 858.8, 540.4, 478.3, 786, and 535 mAh/g at current densities of 0.1, 0.2, 0.5, and 1 A/g, respectively. The capacity retention rate of the FeOOH/GO electrode reached 93.35% after 100 cycles at a high current density of 1 A/g. The excellent electrochemical performance of the FeOOH/GO composite electrode was concluded to be closely related to the addition of GO. To investigate the electrochemical kinetics, electrochemical impedance spectra (EIS) of both samples were collected before cycling. The curve obtained from the test included a semicircle compressed at high frequency and a linear slope at low frequency. In Figure 5F, it shows that the lithium-ion diffusion rate of FeOOH/GO is higher than that of FeOOH in low frequency region. In Figure 5D, the semicircle diameter of the FeOOH electrode was obviously larger than that of the FeOOH/GO composite electrode, indicating that the charge transfer impedance of pure FeOOH was larger than that of the FeOOH/GO composite electrode. In the FeOOH/GO composite electrode materials, the slope of the oblique part in the low frequency region was large. When the slope of the straight line in the low frequency region is larger, the lithium storage by pseudocapacitance becomes more obvious. However, in the pure phase FeOOH electrode material, the angle of the diagonal in the low frequency region was close to 45°, indicating that the electrochemical reaction in the electrode material was a semi-infinite diffusion-controlled reaction process. In other words, the FeOOH/GO composite electrode could not only improve the capacity of lithium storage on the surface but also maintained a fast ion transfer rate; thus, FeOOH/GO had excellent high-rate cycling performance.

**Figure 6:**
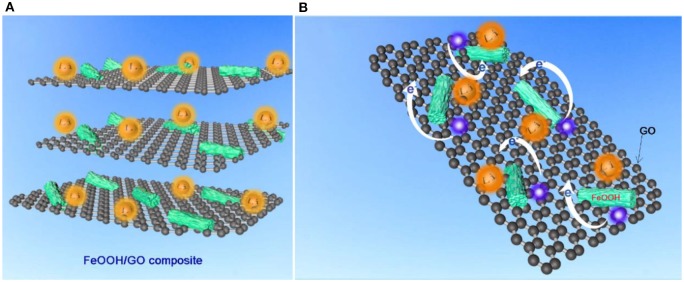
Synergistic electrochemical characteristics of the FeOOH/GO composite of **(A)** the whole frame and **(B)** single layer structure mechanism model.

The excellent electrochemical performance of FeOOH/GO was due to the reticulated graphene structure, as illustrated in Figures 6A,B. First, the FeOOH nanorod structure (edge length ≤ 100 nm) provided a notably short ion diffusion distance and a small volume deformation. Additionally, the graphene network had a small volume deformation during the charging and discharging process, which also helped improve the stability and strength of the overall FeOOH/GO nanostructure, thus avoiding structural collapse due to the occurrence of volume changes during the conversion process. The obtained FeOOH/GO could improve the diffusion rate of lithium ions and significantly reduce the internal resistance of interfacial transfer, thus improving the capacity, rate performance, and cycling stability of the battery. Second, the graphene network was constructed to change the electronic arrangement of the local region and improve the adsorption capacity of ions, thus maximizing the use of space, improving the conductivity and accelerating the ion diffusion rate.

## Conclusions

In this work, FeOOH/GO was synthesized by a hydrothermal method. The FeOOH/GO composite electrode material presents a surface pseudocapacitance-based charge/discharge battery system in the charge discharge process and achieves excellent lithium storage performance. The initial capacity retention of 93.35% can be retained after 100 cycles at 1 A/g. The excellent electrochemistry of the FeOOH/GO composite electrode materials is due to their unique structure, which leads to a pseudocapacitance mechanism of energy storage. When the FeOOH/GO composites contact the electrolyte, the FeOOH grains (<100 nm) shorten the diffusion distance, increase the diffusion rate, and promote the pseudocapacitance mechanism of lithium storage. Another important structural feature is that FeOOH and GO, which are chemically bonded, maintain a close connection. Thus, the chemical bond between FeOOH and GO effectively promotes rapid charge and ion transfer and forms a large number of fast charge transfer channels in the FeOOH/GO composite electrode materials. Compared with other transition metal oxides, this kind of electrode material has a strong competitive advantage. It has a high capacity and stable cycling performance under a high current. Therefore, this kind of electrode material shows high commercial application value.

## Data Availability Statement

The raw data supporting the conclusions of this article will be made available by the authors, without undue reservation, to any qualified researcher.

## Author Contributions

XC, YZ, and ZC contributed to the design and calculation of material. SW, CX, LW, CS, and LL contributed to the synthesis and characterization of material. CZ contributed to data analysis.

## Conflict of Interest

The authors declare that the research was conducted in the absence of any commercial or financial relationships that could be construed as a potential conflict of interest.
